# Enhancement of glucose homeostasis through the PI3K/Akt signaling pathway by dietary with *Agaricus blazei* Murrill in STZ‐induced diabetic rats

**DOI:** 10.1002/fsn3.1397

**Published:** 2020-01-13

**Authors:** Qi Wei, Jie Li, Yishu Zhan, Qiangui Zhong, Baogui Xie, Lei Chen, Bingzhi Chen, Yuji Jiang

**Affiliations:** ^1^ College of Food Science Fujian Agriculture and Forestry University Fuzhou Fujian China; ^2^ Mycological Research Center Fujian Agriculture and Forestry University Fuzhou Fujian China

**Keywords:** *Agaricus blazei* Murrill, metabolomics, PI3K/Akt signaling pathway, type 2 diabetes, UPLC‐MS

## Abstract

*Agaricus blazei* Murrill (ABM) is an edible fungus. This study investigated the protective role of ABM fruiting body against streptozotocin (STZ)‐induced diabetic rats. After 4 weeks of ABM supplement, glucose homeostasis was improved in diabetic rats. Quantitative real‐time and Western blot analyses suggested that ABM could promote the gene and protein expression level of insulin receptor, pyruvate dehydrogenase kinase, phospho‐kinase B, kinase B, phosphatidylinositol 3‐kinase, insulin receptor substrate 1, glucose transporter‐4, and glutamine synthetase, while inhibiting the expression of glycogen synthase kinase 3β and c‐jun N‐terminal kinase 1 and 2. According to multivariate and univariate statistical analysis, liver metabolite profiles of the normal and diabetic rats fed basal and experimental diet were clearly separated. The differential liver metabolites from diabetic rats fed basal and ABM diet‐related pathways including the glycolysis pathway, pentose phosphate pathway, tricarboxylic acid cycle, and oxidative phosphorylation were analyzed. A total of 18 potential biomarker metabolites were identified as differential biomarkers associated with ABM supplement diet.

## Graphical abstract

Intake of *Agaricus blazei* Murrill (ABM) modulated characteristics of diabetic rats. ABM reduced elevated blood glucose and improved hepatic function. ABM plays an important role in ameliorating glucose homeostasis.

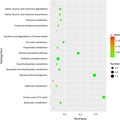

## 1 INTRODUCTION

Type 2 diabetes is characterized by insulin resistance and progressive β cell failure with associated insulin deficiency (Cinti et al., 2016). Irregularly increasing glucose leads to many chronic diseases (Wu, Shi, Wang, & Wang, 2016; Gao, Guo, Qin, Shang, & Zhang, 2017). Thus, the control of blood glucose level is a critical method to prevent the progression of type 2 diabetes and its complications (Martinez, Lockhart, Davies, Lindsay, & Dempster, 2018). In consideration of limitations and negative side effects of commercial chemosynthetic drugs, numerous scientific investigations are being incessantly conducted into various natural products, including triterpenoids (Khanra et al., 2017) and flavonoids (Tanveer, Akram, Farooq, Hayat, & Shafi, 2017), which have a preventive or therapeutic effect on diabetes (Stojkovic et al., 2019; Thompson & Davis, 2017). Therefore, dietary therapies targeting the origin of insulin resistance are preferable for the prevention of diabetes.

*Agaricus blazei* Murrill (ABM) was identified by the Belgain scientist Heinemann in 1967 (Firenzuoli, Gori, & Lombardo, 2008), popularly known as “*Cogumelo do Sol*” in Brazil, “*Himematsutake*” in Japan, or “*Ji Song Rong*” in China, is native to Brazil. ABM is well known as a medicinal mushroom. ABM has been studied in the treatment of cancer, chronic hepatitis, hyperlipidemia, and diabetes. Previous studies have shown that β glucan of ABM played a remarkable role in the beneficial effects of immunological stimulation (Sari, Prange, Lelley, & Hambitzer, 2017). Some other reports indicated the promising antitumor activity of ABM, resulting from its significant potency in stimulating positive responses of the immune system (Huang et al., 2015; Yamanaka et al., 2012). In Japan, ABM was used as the most popular complementary and alternative medicine for cancer patients (Hyodo et al., 2005). Moreover, several studies had illustrated that the ABM has potent hypoglycemic action, which could be useful in the treatment of diabetes mellitus (Yu et al., 2013; Oh et al.,  2010). However, the molecular mechanism of ABM on improving glucose homeostasis in diabetes mellitus, especially in the liver, and the regulating metabolic processes is still unclear. Metabolomics can identify all metabolites in a cell, or a tissue, and it has been applied as an advanced separation and detection method (Zhou, Zhao, Guan, Wang, & Gao, 2017; Zhang, Sun, Yan, Wang, & Wang, 2016; Benchoula et al., 2019). Ultra‐performance liquid chromatography–mass spectrometry (UPLC‐MS) has been widely used to obtain metabolite changes and the related biochemical pathways. Metabolomics may elucidate many physiological processes (Theodoridis, Gika, & Wilson, 2008; Zhou et al., 2015). The nontargeted metabolomics usually uses a full scan mode to fully detect all metabolites in the sample, with an unbiased fashion. The changes in different samples can be detected during the disease development. Thus, it can provide a global view of dynamic metabolic variations. Nontargeted metabolomics is helpful for discovering new potential metabolites and new metabolic pathways, which are important for the discovery of key biomarkers and disease diagnosis (Naz, Vallejo, Garcia, & Barbas, 2014).

The liver is the major organ to regulate glucose metabolism by many intricate signaling pathways (Huang, Chang, Wu, Shih, & Shen, 2016). Phosphatidylinositol 3‐kinase (PI3K)/kinase B (Akt) pathway is one of the key signal pathways, which is believed as a major mechanism involved in the development of insulin resistance (Gao et al., 2015). However, the research about the molecular mechanism of ABM in hypoglycemia and hepatic insulin resistance in diabetic rats is limited. The purpose of this study was to investigate the molecular mechanism of ABM on improving glucose homeostasis in Streptozotocin (STZ)‐induced rats through the PI3K/Akt signaling pathways and screen out potential metabolite biomarkers from diabetic rats with ABM supplement using UPLC‐MS system. These results may contribute to a better understanding of the protective role of ABM in diabetic rats.

## 2 MATERIALS AND METHODS

### 2.1 Chemicals

Streptozotocin was obtained from Sigma. UNlQ‐10 Column Trizol Total RNA Isolation Kit, 4S Red Plus Nucleic Acid Stain, and Agarose were purchased from Sangon Biotech Co., Ltd. Antibodies such as PI3K, phospho‐kinase B (p‐Akt), Akt, glycogen synthase kinase 3β (GSK3β), glucose transporter‐4 (GLUT4), insulin receptor substrate 1 (IRS1), and glutamine synthetase (GS) were purchased from Ruiying Biological Co., Ltd. Secondary antibodies were bought from Sangon Biotech Co., Ltd. Methanol, acetonitrile, and formic acid were purchased from CNW Technologies GmbH. L‐2‐chlorophenylalanine was purchased from Shanghai Hengchuang Bio‐technology Co., Ltd. All the remaining reagents used in this experiment were of analytical grade.

### 2.2 Plant material and extraction

The samples of ABM (dried product) were purchased from Gutian Shenger Food Co., Ltd. The dried samples were crushed and filtered through 80‐mesh screen. The ABM powder was mixed with pure ethanol (1:30) and extracted at 70°C for 3 hr. The extraction process was repeated twice. After extraction, the supernatant was filtrated through a Whatman no. 2 paper and then concentrated to dryness by evaporating with a rotary evaporator (N‐1100; Eyela) and water bath (SB‐1100; Eyela) to remove ethanol. The residue was collected as ethanol extract of ABM. Besides, the ethanol extract was extracted three times with ethyl acetate at room temperature for 2 hr with shaking. The ethyl acetate fraction was finally evaporated to dryness at 45°C as the ethyl acetate extract of ABM. The extracts were stored at −20°C until use.

### 2.3 Animals

Eight‐week‐old male Sprague Dawley (*SD*) rats were supplied by Fuzhou General Hospital of Nanjing Military Command Animal Center, and maintained in a controlled room with specific‐pathogen‐free, constant temperature (23 ± 2°C) and humidity (50 ± 10%), and 12‐hr light/dark cycle (lights on at 8 a.m.) at the Animal Experiment Center of Fuzhou General Hospital of Nanjing Military Command. All rats were fed normal chow and kept free access to water and food during the experiment. The Animal Ethics Committee of Fuzhou General Hospital of Nanjing Military Command approved all experiments.

### 2.4 Experimental procedure

Forty‐two male *SD* rats were allowed to adapt to the new environment for 7 days and then were divided into seven groups randomly: normal control group (N group), diabetes control group (M), positive control group (P), treated with ethanol extract from ABM (EH and EL), and treated with ethyl acetate extract from ABM (AH and AL). N, M, P, EH, EL, AH, and AL groups were treated with aqueous solution, 0.1 mg/kg body weight (bw) of glimepiride, 500 mg/kg bw ethanol extract from ABM, 250 mg/kg bw ethanol extract from ABM, 500 mg/kg bw ethyl acetate extract from ABM, and 250 mg/kg bw ethyl acetate extract from ABM once a day for 28 days, respectively. On the first day, N group comprised of six rats were injected with 100 mmol/L citrate buffer (pH = 4.5), and the other groups comprised of thirty‐six rats received 55 mg/kg bw dose injection of STZ freshly dissolved in 100 mmol/L citrate buffer. After 48 hr, post‐STZ injection, diabetic rats were identified by measuring fasting blood glucose levels. Rats with a fasting blood glucose level above 11.1 mmol/L were selected as diabetic rats for experiment. The glimepiride and different doses of sample were administered in aqueous solution (3%, v/v Tween 80 in water) once a day.

### 2.5 Tissue sample collection

At the end of the 4‐week experiment period, the 12‐hr fasted rats were anesthetized (5 ml/kg bw chloral hydrate) and sacrificed. The liver was quickly removed and rinsed in saline solution to remove the blood and immediately frozen and stored at −80°C for various analyses.

### 2.6 Oral glucose tolerance test

Oral glucose tolerance test (OGTT) was performed in 12‐hr fasted rats from each group. Vehicle (distilled water) was orally administered to N and M groups. Doses of 250 and 500 mg/kg ethanol or ethyl acetate extract from ABM were orally administered to EL, EH, AL, and AH groups, respectively. After 30 min, all rats were orally treated with glucose load at a dose of 2 g/kg body weight. Blood was taken from the tip of the tail at 0, 30, 60, and 120 min from all groups to obtain the blood glucose content using a blood glucose meter (HGM‐121; Omron). Area under the curve (AUC) of oral glucose tolerance was used to measure the glucose concentration over time.

### 2.7 Quantitative Real‐Time PCR analysis

Total RNA from liver tissue was extracted by RNA extraction kit (Sangon Biotech) in accordance with the manufacturer's instructions. The concentration of the total RNA was determined by the ultraviolet spectrophotometer. The cDNA was synthesized using cDNA synthesis kit (Thermo) according to the instructions provided by the manufacture. The quantitative real‐time PCR (qRT‐PCR) analysis was performed with SG Fast qPCR Master Mix (Sangon Biotech) in a real‐time detector (ABI). The reaction mixtures were incubated at 95°C for 3 min, followed by 45 cycles of incubation at 95°C for 7 s at primer‐specific annealing temperatures and then 72°C for 15 s. The sequences of the primers in this study are shown in Table S1. The glyceraldehyde 3‐phosphate dehydrogenase gene was used as an internal control gene. The gene relative expression level was analyzed by 2^−△△Ct^ method.

### 2.8 Western blot analysis

Protein of liver tissue was extracted with RIPA buffer and then centrifuged at 10,000 Χ *g* for 10 min at 4°C. The total protein was determined by BCA protein assay kit (Sangon Biotech). Subsequently, the protein of liver was mixed with buffer and then boiled for 10 min. Equal amount of each protein was subjected to sodium dodecyl sulfate–polyacrylamide gel electrophoresis for 0.5 hr at 80 V, followed by 2.5 hr at 100 V. The protein samples were subsequently transferred to a PVDF membrane (Merck Millipore) and then blocked with 5% skim powered milk in Tris‐buffered saline and Tween 20 (TBST, Sangon Biotech) for 2 hr at room temperature in a shaker. Incubation of the primary antibodies was carried out at 4°C overnight for PI3K, p‐Akt, Akt, IRS1, GLUT4, GS, and GSK3β, followed by incubation with the corresponding secondary antibodies. The membrane was exposed to enhanced chemiluminescent reagents according to the manufacturer's instructions. The Tannon imaging system (Tannon) was used to detect protein bands, and the intensity of bands was quantified by Image J software.

### 2.9 Liver metabolomics

#### 2.9.1 Sample preparation

Thirty milligrams accurately weighed liver sample was transferred to a 1.5‐ml Eppendorf tube with 20 µl internal standard (2‐chloro‐l‐phenylalanine in methanol, 0.3 mg/ml) and 400 µl ice‐cold methanol/water (4/1, v/v). Samples were stored at −80°C for 2 min and then grinded for 2 min, ultrasonicated at 60 Hz for 10 min, and then the samples stored at −20°C for 30 min. The extract was centrifuged at 10,000 Χ *g*, 4°C for 15 min. The supernatants (200 µl) from each tube were collected using crystal syringes, filtered through 0.22‐µm microfilters and transferred to UPLC vials. The vials were stored at −80°C until UPLC‐MS analysis. Quality control (QC) samples were prepared by mixing aliquots of the all samples to be a pooled sample.

#### 2.9.2 Method development and validation

An ACQUITY UPLC system (Waters Corporation) coupled with an AB SCIEX Triple TOF 5600 System (AB SCIEX) was used to analyze the metabolic profiling in both ESI‐positive and ESI‐negative ion modes. An ACQUITY UPLC BEH C18 column (1.7 μm, 2.1 × 100 mm) was employed in both positive and negative modes. The binary gradient elution system consisted of (A) water (containing 0.1% formic acid, v/v) and (B) acetonitrile (containing 0.1% formic acid, v/v), and separation was achieved using the following: 5%–20% B over 0–2 min, 20%–60% B over 2–4 min, 60%–100% B over 4–11 min, the composition was held at 100% B for 2 min, then 11–13 min, 100% to 5% B, and 13.5–14.5 min holding at 5% B. The flow rate was 0.4 ml/min. The column temperature was 45°C. All the samples were kept at 4°C during the analysis. The injection volume was 5 μl.

Data acquisition was performed in full scan mode (m/z ranges from 70 to 1,000) combined with IDA mode. Parameters of mass spectrometry were as follows: ion source temperature, 550°C (+) and 550°C (−); ion spray voltage, 5,500 V (+) and 4,500 V (−); curtain gas of 35 PSI; declustering potential, 100 V (+) and −100 V (−); collision energy, 10 eV (+) and −10 eV (−); and interface heater temperature, 550°C (+) and 600°C (−). For IDA analysis, range of m/z was set as 50–1000, and the collision energy was 30 eV. The QC samples were injected at regular intervals (every 10 samples) throughout the analytical run to provide a set of data from which repeatability can be assessed.

#### 2.9.3 Data processing and statistical analysis

The raw data were converted to common data format files using a conversion software program MSconventer. Metabolomics data were acquired using the software XCMS 1.50.1 version, which produced a matrix of features with the associated retention time, accurate mass, and chromatography. The metabolite ions which had less than 30% RSD were used for the further data processing.

The UPLS‐MS data were imported into the SIMCA software package (version 14.0, Umetrics). Principle component analysis (PCA) and (orthogonal) partial least‐squares discriminant analysis (OPLS‐DA) were carried out to visualize the metabolic alterations among experimental groups, after mean centering and unit variance scaling. Variable importance in the projection (VIP) ranks the overall contribution of each variable to the OPLS‐DA model, and those variables with VIP >1 are considered relevant for group discrimination. In this study, the default 7‐round cross‐validation was applied with 1/7 of the samples being excluded from the mathematical model in each round, in order to guard against overfitting.

The differential metabolites were selected on the basis of the combination of a statistically significant threshold of VIP values obtained from the OPLS‐DA model and p value from a two‐tailed Student's *t* test on the normalized peak areas, where metabolites with VIP >1 and *p* < .05 were included, respectively. In addition, fold change analysis (FC analysis) was also conducted in the univariate analysis. The database built by Dalian Institute of Chemical Physics, Chinese Academy of Sciences and Dalian Chem Data Solution Information Technology Co., Ltd, The Human Metabolome Database (HMDB, http://www.hmdb.ca/), and METLIN (https://metlin.scripps.edu/) were used to identify the metabolites. The Kyoto Encyclopedia of Genes and Genomes (KEGG, http://www.kegg.jp/) was used for metabolic pathways analysis.

## 3 RESULTS

### 3.1 ABM improved OGTT of diabetic rats

**Figure 1:**
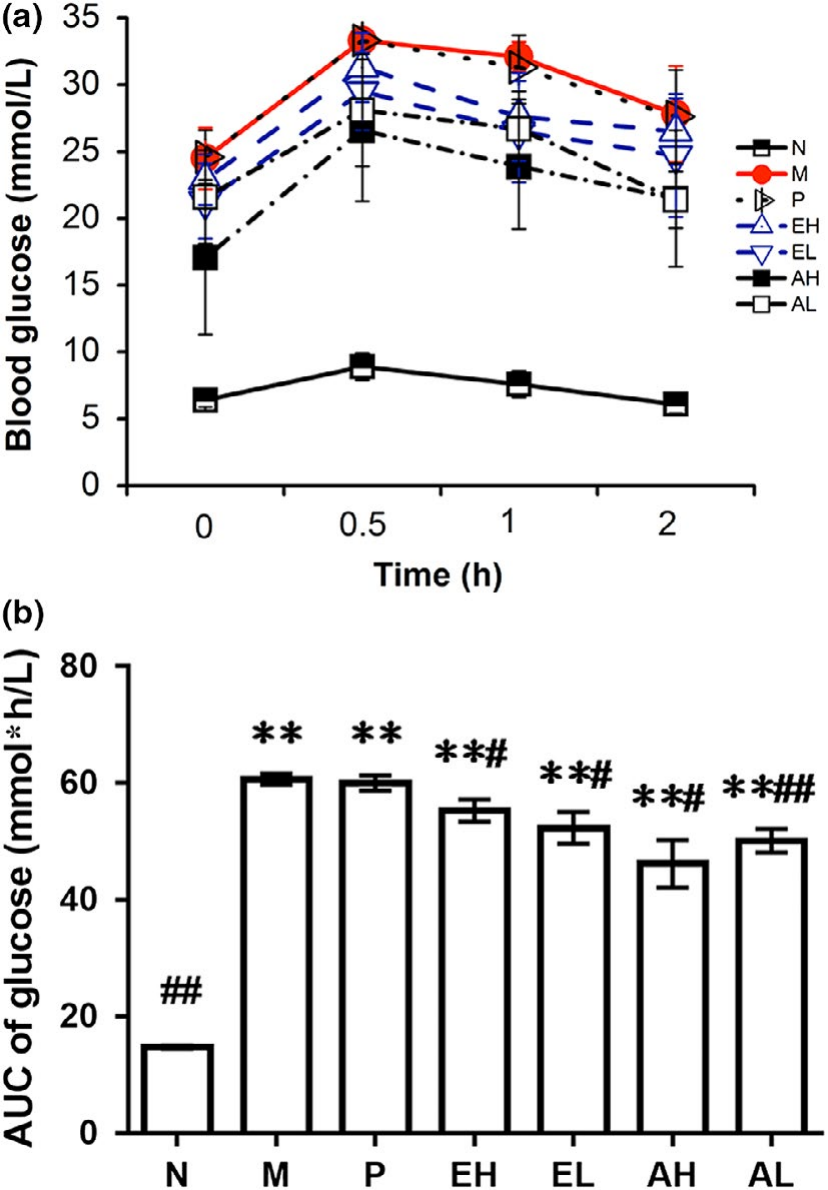
Effects of *Agaricus blazei* Murrill extracts on oral glucose tolerance test (OGTT) and AUC in streptozotocin ‐induced mice. (a) OGTT at day 21, (b) AUC at day 21. Data are expressed as the mean ± *SD* for six rats in each group. Compare with normal control group, *p＜.05, **p＜.01; compare with diabetes control group, #*p*＜.05, ##*p*＜.01. AUC, area under the curve

After 3 weeks of supplement with ABM, 12‐hr fasted rats were subjected to an OGTT. As shown in Figure 1a, the blood level of M group rapidly increased by oral supplementation of glucose (2 g/kg) within 30 min, and it remained at a high level over the next 90 min. The blood glucose level increased to the peak in all groups, but it dropped to the initial level after 120 min in N group. After oral glucose administration, the blood level of diabetic rats was decreased by treated with ABM in comparison with M group. OGTT areas under the glucose curve (AUC) over 120 min were used for evaluating the glucose tolerance for diabetic rats. The AUC of ABM‐treated groups markedly decreased compared to group M (Figure 1b, *p* < .05), which suggested that ABM could improve the glucose tolerance in diabetic rats.

### 3.2 ABM improved glucose homeostasis through the PI3K/Akt pathway

**Figure 2:**
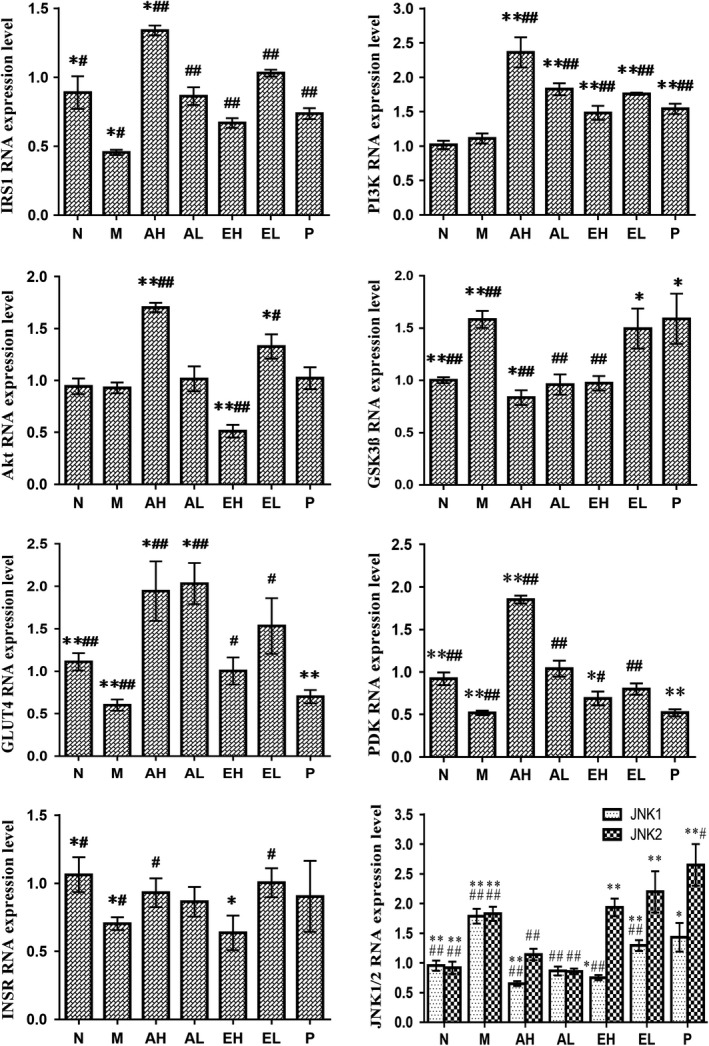
Effect of *Agaricus blazei* Murrill extracts on the gene expression in the PI3K/Akt pathway. Compare with normal control group, *p＜.05, **p＜.01; compare with diabetes control group, #*p*＜.05, ##*p*＜.01

In this investigation, the hepatic insulin signaling‐related gene expression of insulin receptor (INSR), IRS1, PI3K, pyruvate dehydrogenase kinase (PDK), Akt, GSK3β, c‐jun N‐terminal kinase 1 and 2 (JNK1/2), and GLUT4 were evaluated by qRT‐PCR, inquiring into the molecular mechanism of ABM by improving glucose homeostasis. As shown in Figure 2, ABM‐treated groups increased the gene expression of INSR, IRS1, PI3K, PDK, Akt, and GLUT4, compared to the M group. The gene expression levels of JNK1/2 and GSK3β (*p* < .05) significantly increased in the M group compared to N group. Moreover, treatment with ABM showed a reverse of the gene expression levels of JNK1/2 and GSK3β. The expression of hepatic insulin signaling‐related genes in AH group was basically higher than AL group. While compared to the EL group, the expression levels of these genes in EH group were decreased.

**Figure 3:**
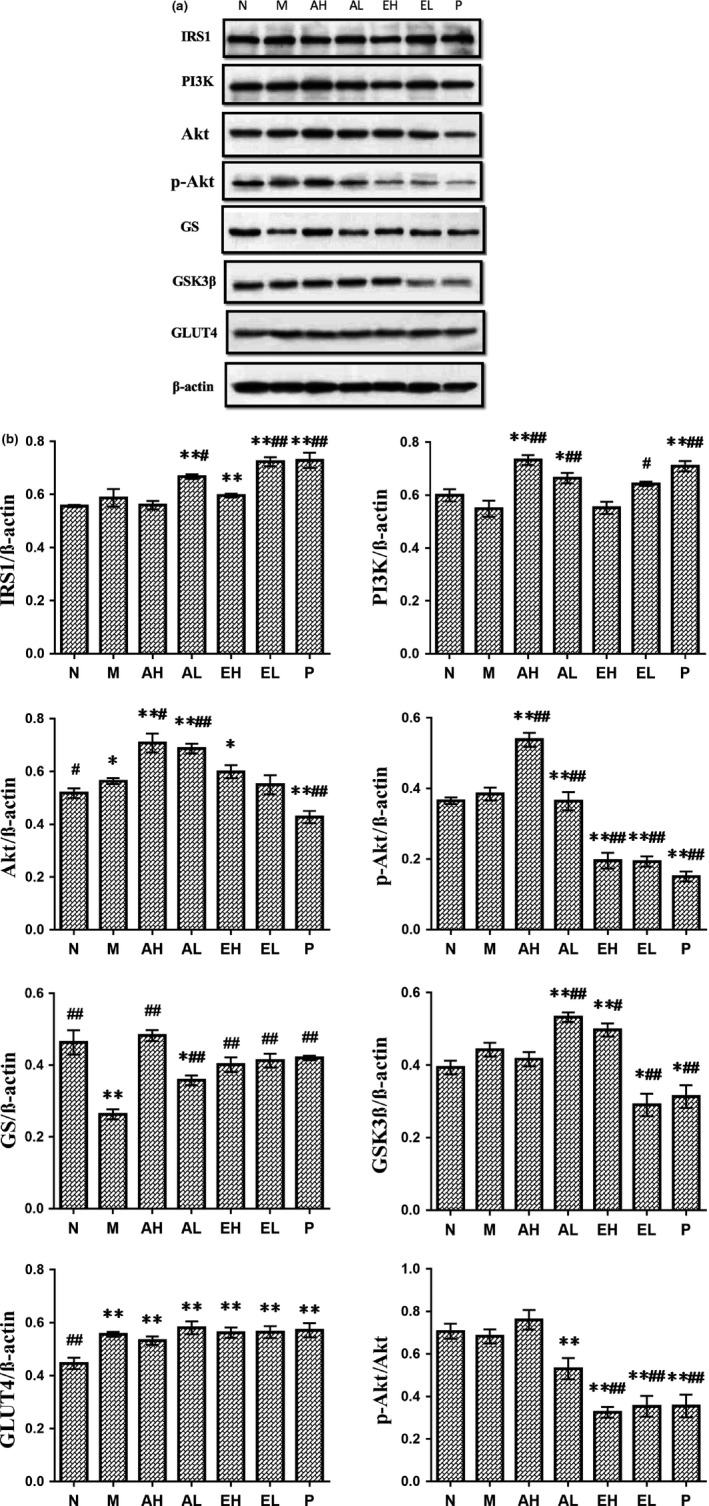
Western blot was used to express the effect of *Agaricus blazei* Murrill extracts on the key proteins in the PI3K/Akt pathway. Compare with normal control group, *p＜.05, **p＜.01; compare with diabetes control group, #*p*＜.05, ##*p*＜.01

The effects of ABM on the expression of the main proteins in PI3K/Akt signaling pathway were investigated through Western blot. The results in Figure 3 indicated that the ABM could increase the expression of IRS1, PI3K, and GLUT4 in STZ‐induced rats. Akt and p‐Akt were measured to determine whether PI3K downstream signaling was affected by ABM in diabetic rats. As demonstrated in Figure 3, AH group showed a significant increase in the protein expression of Akt and p‐Akt in liver tissue compared to M group (*p* < .05). To investigate the molecular mechanism of ABM on glycogen synthesis, we evaluated the level of GS protein and GSK3β protein (Figure 3). The protein expression of GS was associated with a significant increase in ABM‐treated groups (*p* < .05), whereas the protein expression of GSK3β was decreased. The results showed that administration of ABM significantly enhanced the expression of the main genes and proteins in the insulin‐resistant pathway and then indicated that ABM had a positive effect on amelioration of insulin resistance through the PI3K/Akt signaling pathways.

### 3.3 Metabolic profiles

Western blot and qRT‐PCR results showed that AH group was associated with a significant effect on enhancing glucose homeostasis. Thus, the N, M, and AH groups were chosen for further metabolomics profiling. The metabolic profiling of liver samples from N, M, and AH groups was analyzed by UPLC‐MS system in ESI‐positive and ESI‐negative ion modes. The endogenous metabolites of the three groups were well separated (Figure S1). A total of 2,102 metabolite ions were acquired in positive ion mode, and 2,910 metabolite ions were acquired in negative ion mode. 45.91% of ions in positive ion mode and 61.61% in negative ion mode exhibited less than 30% of RSD, which displayed good reproducibility of the metabolomics method. The metabolite ions which had less than 30% of RSD were used for the further data processing. Therefore, the UPLC‐MS system repeatability and stability were deemed acceptable.

**Figure 4:**
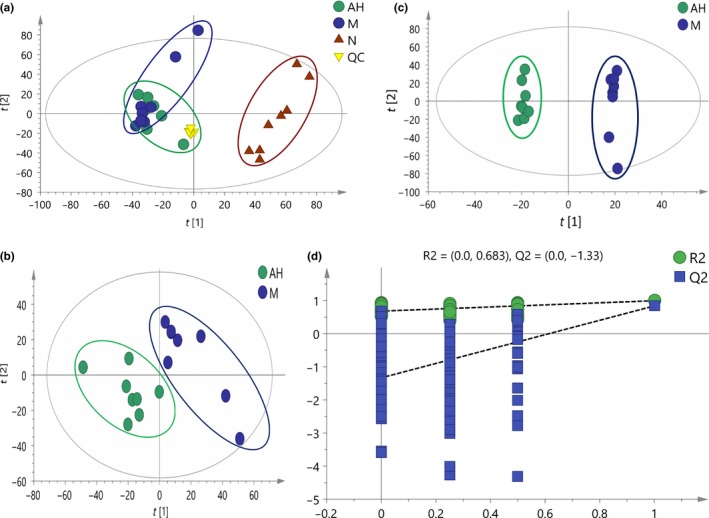
Multivariate statistical score graph. (a) PCA, (b) PLS‐DA, (c) OPLS‐DA, (d) permutation test. OPLS‐DA, partial least‐squares discriminant analysis; PCA, principle component analysis

Using multivariate analysis, PCA and OPLS‐DA were used to acquire normalized data. Three clusters from different groups were shown in the PCA score plot. A clear separation of group N was observed in Figure 4a. Group M showed a loose clustering than group N. Group M and group AH were closer to each other. OPLS‐DA was used to gain better discrimination among the group M and group AH. Group M and group AH were separated clearly in Figure 4b,c, which suggested that substantial biochemical changes occurred in these two groups.

### 3.4 Metabolic pathway analysis of identified differential metabolites

**Figure 5:**
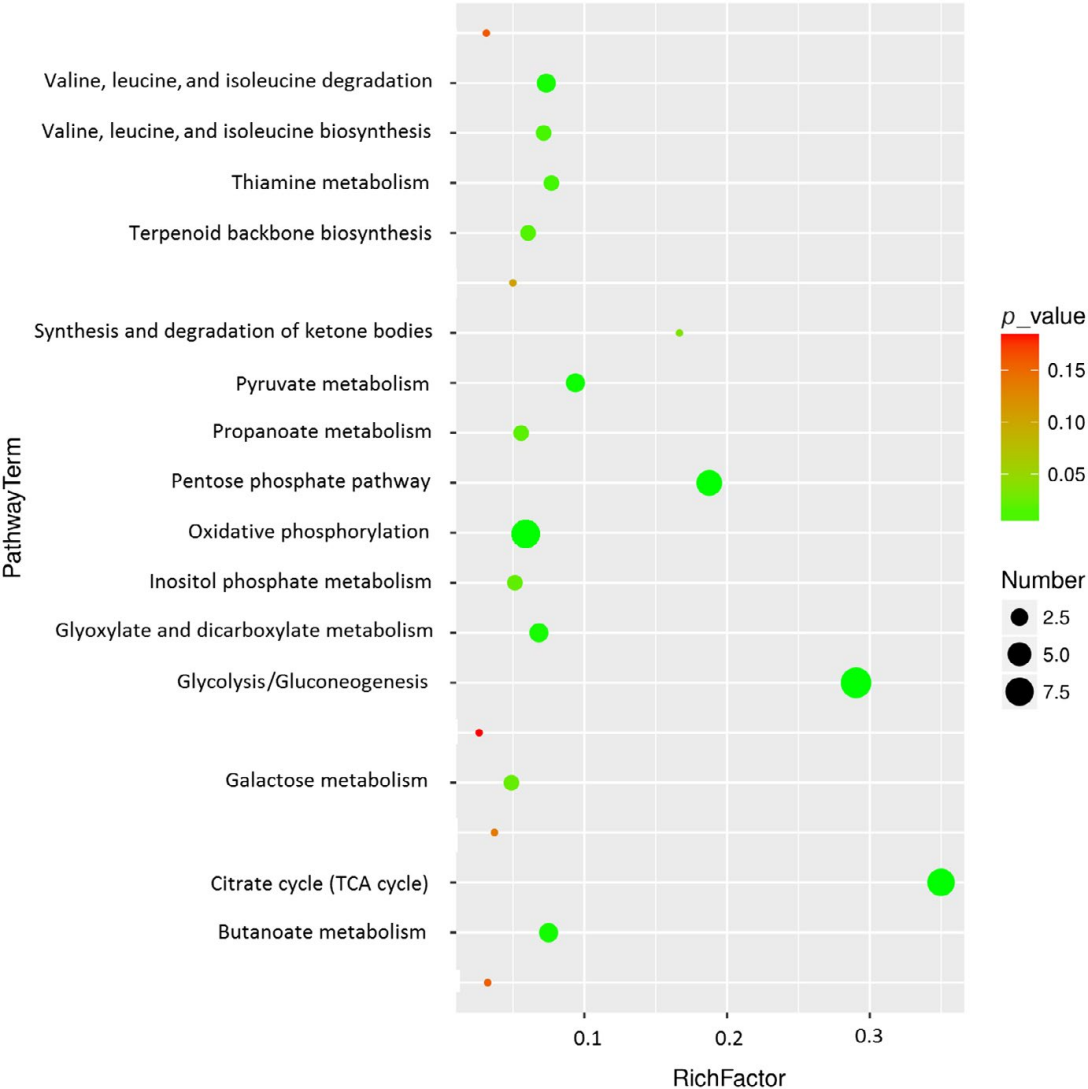
Effects of *Agaricus blazei* Murrill extract on metabolic pathways in diabetic rats

As shown in Table 1, there are 18 potential biomarker metabolites found between group M and group AH. Based on KEGG results, 31 metabolic pathways were detected in the liver samples when compared with M group and AH group. Moreover, 15 metabolic pathways, including oxidative phosphorylation (1), glycolysis/gluconeogenesis (9), citrate cycle (TCA cycle) (7), pentose phosphate pathway (7), pyruvate metabolism (3), butanoate metabolism (2), valine, leucine, and isoleucine degradation (2), glyoxylate and dicarboxylate metabolism (5), thiamine metabolism (2), valine, leucine, and isoleucine biosynthesis (1), terpenoid backbone biosynthesis (2), propanoate metabolism (3), inositol phosphate metabolism (2), galactose metabolism (2), and synthesis and degradation of ketone bodies (1), were significantly influenced by the supplement with ABM (Figure 5, Table S2).

**Table 1:** Different metabolites in diabetic rats fed basal and experimental diet for 4 weeks

Metabolites	M/Z	Rt (min)	FC
NAD	709.1149	0.73	3.09
2‐Phospho‐D‐glycerate	218.0026	0.6	0.89
D‐Ribose 1,5‐bisphosphate	309.0886	0.63	1.12
Alpha‐D‐Ribose 1‐phosphate	265.0828	1.17	1.10
cis‐Aconitate	219.105	1.34	1.05
3‐Phospho‐D‐glyceroyl phosphate	242.9868	4.04	0.75
Citrate	237.1185	8.56	0.96
Salicin	321.2421	9.22	2.22
Acetyl‐CoA	844.5506	12.85	0.88
D‐Glyceraldehyde 3‐phosphate	193.0486	0.57	1.04
Alpha‐D‐Glucose 6‐phosphate	261.1432	1.79	0.69
Thiamin diphosphate	448.3053	4.11	0.92
Arbutin 6‐phosphate	335.2271	4.2	0.83
2‐Amino‐2‐deoxy‐D‐gluconate	233.1215	4.22	0.86
2‐(alpha‐Hydroxyethyl) thiamine diphosphate	470.3789	4.62	1.02
Arbutin 6‐phosphate	335.2253	5.28	0.94
3‐Carboxy‐1‐hydroxypropyl‐ThPP	550.386	7.68	0.96
Succinyl‐CoA	868.6039	11.38	0.84

Abbreviations: FC, fold change; Rt (min), retention time.

## 4 DISCUSSION

Type 2 diabetes is characterized by insulin resistance, which is associated with hyperglycemic. The maintenance of glucose homeostasis is a complex process in which glucose uptake and production play a major role (Buse, 2011; Jiang et al., 2014). The liver plays a critical role in regulating glucose metabolism, including glycolysis, gluconeogenesis, glycogenolysis, and glycogenesis (Srinivasan, Viswanad, Asrat, Kaul, & Ramarao, 2005). STZ‐induced diabetic rats were selected as in vivo model because which were more easily to develop pathological features resembling type 2 diabetes mellitus (Båvenholm, Pigon, Östenson, & Efendic, 2001; Srinivasan & Ramarao, 2007).

On the basis of the results, ABM showed the improvement of oral glucose tolerance in diabetic rats. These results proved the positive effects of ABM supplement diet on relieving hyperglycemia in diabetic rats. Previous studies have showed that the PI3K/Akt pathway plays a significant role in metabolic effect on insulin. Akt is the major downstream target of PI3K signaling pathway. It facilitates glucose uptake and glycogen synthesis in the liver (Gandhi et al., 2013; Huang, Liu, Guo, & Su, 2018). To better understanding the molecular mechanism underlying the effects of ABM supplement diet on diabetic rats, we investigated the phosphorylation of Akt, which is the key molecular mediating the metabolic effects of insulin signaling. Figure 3 suggests AH group was associated with significant increase of the level of Akt and p‐Akt compared with the M group (*p* < .05). In addition, tyrosine phosphorylation of IRS1 can activate the PI3K pathway to induce phosphorylation of Akt, which is an important mediator related to insulin's downstream metabolic actions. GSK3β activity can be inactivated by insulin signaling through insulin receptor IRS1, PI3K, and the action of Akt to phosphorylate specific serine residues on the enzyme (Wang et al., 2017, 2012). Therefore, the activation of Akt leads to inactivation of GSK3β, which in turn enhances the expression of GS protein, potentially, on glucose transport activity.

The results of qRT‐PCR and Western blot analyses showed that Akt, p‐Akt, GS, IRS1, GLUT4, and PI3K was downregulated, whereas JNK1/2 and GSK3β was significantly elevated in the M group (*p* < .05). The AH group showed a significant elevation of p‐Akt (*p* < .05) and lower the level of GSK3β were observed. Subsequently, the qRT‐PCR data confirmed that the dietary supplement with ABM could reverse the increase of JNK1/2. In addition, the increased protein expression of GS in the ABM‐treated rats was observed, suggesting that the glycolysis in diabetic rats was improved.

Metabolomics is a highly beneficial approach to assess the creature physiological status. Through metabolomics analysis among normal rats, diabetic rats fed basal diet and diabetic rats fed with experimental diet, the potential biomarkers between the different groups were deceptively detected. Overall, liver metabolite profiles of normal rats were visibly separated with diabetic rats. After 4 weeks with ABM supplement diet, metabolite profiles of group AH were much more different from group M. A total of 18 metabolites were identified as potential biomarker metabolites associated with ABM supplement diet. NAD, alpha‐D‐Glucose 6‐phosphate, 3‐Phospho‐D‐glyceroyl phosphate, thiamine diphosphate, 2‐Phospho‐D‐glycerate, D‐Glyceraldehyde 3‐phosphate, and 2‐(alpha‐Hydroxyethyl) thiamine diphosphate were involved in the glucose metabolism. Thus, it indicated that NAD, alpha‐D‐Glucose 6‐phosphate, 3‐Phospho‐D‐glyceroyl phosphate, thiamine diphosphate, 2‐Phospho‐D‐glycerate, D‐Glyceraldehyde 3‐phosphate, and 2‐(alpha‐Hydroxyethyl) thiamine diphosphate were responsible for the antidiabetic effect in rat models.

NAD is involved in many physiological activities such as cellular material metabolism, energy synthesis, and cellular DNA repair. The pathogenesis of type 2 diabetes and its complications are closely related to the state of oxidative stress. To date, it has been demonstrated that long‐term hyperglycemia by diabetes could induce the generation of advanced glycation end products, which are associated with the generation of reactive oxygen species, indicating the oxidative damage may be the underlying mechanism for hyperglycemia (Teng & Chen, 2017). Interestingly, the results revealed that oxidative phosphorylation was inhibited; consequently, the body metabolism and energy synthesis were slowed down in diabetic rats. After 4 weeks of supplement diet with ABM, the level of NAD in oxidative phosphorylation metabolic pathway was improved in diabetic rats. In the meantime, the levels of Glyceraldehyde‐3P and 2‐Hydroxyethyl‐ThPP in the pathway of glucose metabolism were upregulated.

The metabolic pathways analysis indicated that 15 metabolic pathways showed significant differences between group M and group AH (*p* < .05). Glycolysis, pentose phosphate pathway, tricarboxylic acid cycle (TCA), and oxidative phosphorylation were significant interference among the 15 metabolic pathways. Consequently, we assumed that the ABM diet‐related pathways have great alterations in diabetic rats.

In conclusion, we confirmed that ABM could activate PI3K/Akt signaling pathways and improve glucose homeostasis. The analysis of metabolomics suggested that the metabolic alterations associated in the ABM supplement diet are reflected in the liver tissues. Thus, we suspect that ABM had therapeutic potential for the therapy for type 2 diabetes mellitus. The present study offers valuable information for understanding the molecular mechanism of ABM in ameliorating type 2 diabetes mellitus and provides key data for the future development of functional food and dietary supplements, once sufficient further research on ABM has been performed.

## 5 CONCLUSIONS

In summary, we demonstrated that ABM successfully improved glucose homeostasis in STZ‐induced diabetic rats. Results from the qRT‐PCR and Western blot analysis provided evidences that the PI3K/Akt signaling pathway in diabetic rats was effectively activated over 4 weeks of ABM supplement. A total of 18 metabolites were identified as differential biomarkers associated with ABM supplement diet. It suggested that ABM elicits protective effects against the STZ‐induced diabetic rats. These findings may contribute to a better understanding of the beneficial effect of ABM as health foods and remedy for diabetes in the future.

## CONFLICT OF INTEREST

The authors declare that they do not have any conflict of interests.

## ETHICAL APPROVAL

This study was approved by the Animal Ethics Committee of Fuzhou General Hospital of Nanjing Military Command in China.
