# Supplementary material for: Enhancement of glucose homeostasis through the PI3K/Akt signaling pathway by dietary with Agaricus blazei Murrill in STZ‐induced diabetic rats
Source: Food Sci Nutr. 2020 Jan 13;8(2):1104–14. doi: 10.1002/fsn3.1397 (PMC7020295; doi:10.1002/fsn3.1397)

**Supplement tables**

Table 1S Designed primer set for qRT-PCR.

| Gene | Sequence (5'-3') | Size (bp) |
| --- | --- | --- |
| INSR-F | GTTTGCCCAACCATCTGTAAGT | 102 |
| INSR-R | CTTGGTAGGGTCATCGGGTT |  |
| IRS1-F | GCCATGAGCGATGAGTTTC | 107 |
| IRS1-R | GGCGGAGGATTGTTGAGAT' |  |
| JNK1-F | CTCCAGCACCCGTACATCAA | 164 |
| JNK1-R | CCATTCTTAGTTCGCTCCTCC |  |
| JNK2-F | CAAGGGATTGTTTGTGCTGC | 83 |
| JNK2-R | TGGTTCTGAAAAGGACGGCT |  |
| PI3K-F | CAGTAGGCAACCGTGAAGAAA | 214 |
| PI3K-R | GGTGAAGATTCTACATTTGGAGG |  |
| AKT-F | AGGCATCCCTTCCTTACAGC | 161 |
| AKT-R | GACACAATCTCCGCACCGTA |  |
| GSK3β-F | TCCTTATCCCTCCTCACGCT | 104 |
| GSK3β-R | GTTATTGGTCTGTCCACGGTCT |  |
| PDK1-F | CAGACAAAGGCGTTTATCCC | 199 |
| PDK1-R | AATCCGTAACCAAATCCAGC |  |
| GLUT4-F | TTCCTTCTATTTGCCGTCCTC | 145 |
| GLUT4-R | TACTGGGTTTCACCTCCTGCT |  |
| GAPDH -F | TGGGTGTGAACCATGAGAAGT | 156 |
| GAPDH -R | TGAGTCCTTCCACGATACCAA |  |

Table 2S The remarkable potential metabolomics biomarkers and pathways in diabetic rats fed basal and experimental diets.

| Pathways | Metabolites |
| --- | --- |
| Oxidative phosphorylation | NAD |
| Glycolysis / Gluconeogenesis | alpha-D-Glucose 6-phosphate, Arbutin 6-phosphate, Salicin, D-Glyceraldehyde 3-phosphate, ThPP, 2-Hydroxyethyl-ThPP, Acetyl-CoA, 2-Phospho-D-glycerate, Glycerate-1,3P_2_ |
| Citrate cycle (TCA cycle) | ThPP, 2-Hydroxyethyl-ThPP, Acetyl-CoA, Citrate, cis-Aconitate, Succinyl-CoA, 3-Carboxy-1-hydroxypropyl-ThPP |
| Pentose phosphate pathway | D-Glyceraldehyde 3-phosphate, 2-Phospho-D-glycerate, D-Ribose 1,5-bisphosphate, alpha-D-Ribose 1-phosphate, alpha-D-Glucose 6-phosphate, D-Glucosaminate |
| Pyruvate metabolism | ThPP, 2-Hydroxyethyl-ThPP, Acetyl-CoA |
| Butanoate metabolism | Succinyl-CoA, Acetyl-CoA |
| Valine, leucine and isoleucine degradation | Acetyl-CoA, ThPP |
| Glyoxylate and dicarboxylate metabolism | Citrate, Succinyl-CoA, Acetyl-CoA, cis-Aconitate, 2-Phospho-D-glycerate |
| Thiamine metabolism | D-Glyceraldehyde 3-phosphate, Thiamin diphosphate |
| Valine, leucine and isoleucine biosynthesis | Acetyl-CoA |
| Terpenoid backbone biosynthesis | Acetyl-CoA, D-Glyceraldehyde 3-phosphate |
| Propanoate metabolism | ThPP, Acetyl-CoA, Succinyl-CoA |
| Inositol phosphate metabolism | D-Glyceraldehyde 3-phosphate, Acetyl-CoA |
| Galactose metabolism | alpha-D-Glucose 6-phosphate, D-Glyceraldehyde 3-phosphate |
| Synthesis and degradation of ketone bodies | Acetyl-CoA |

**Supplement figure**

**Figure 1S** Representative total ion current (TIC) chromatograms of mice liver tissues in (N) normal control group, (M) diabetes control group, (AH) 500 mg/kg bw ethyl acetate extract from *Agaricus blazei* Murrill; (1) positive ion mode, (2) negative ion mode.

**Figure 1S**


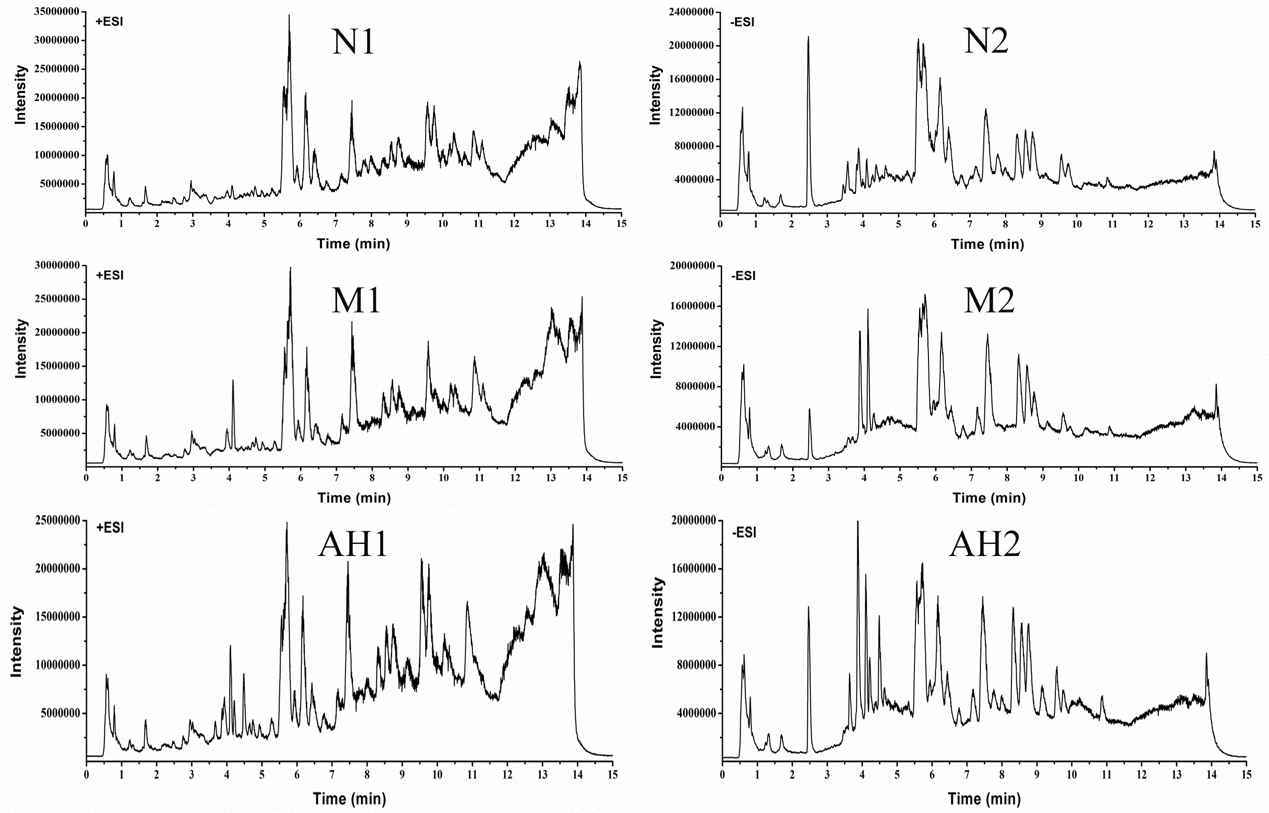

Supplement: Supplementary file 1 [file FSN3-8-1104-s001.docx]
